# Draft Genome Sequence of Shewanella sp. Strain CP20

**DOI:** 10.1128/genomeA.00256-15

**Published:** 2015-04-09

**Authors:** Carla Lutz, Qi Xiang Martin Tay, Shuyang Sun, Diane McDougald

**Affiliations:** a Centre for Marine Bio-Innovation, School of Biotechnology and Biomolecular Science, University of New South Wales, Sydney, Australia; b Singapore Centre on Environmental Life Sciences Engineering, Nanyang Technological University, Singapore; c School of Biological Sciences, Nanyang Technological University, Singapore; d ithree Institute, University of Technology, Sydney, Sydney, Australia

## Abstract

Shewanella sp. CP20 is a marine bacterium that survives ingestion by *Tetrahymena pyriformis* and is expelled from the protozoan within membrane-bound vacuoles, where the bacterial cells show long-term survival. Here, we report the draft genome sequence of Shewanella sp. CP20 and discuss the potential mechanisms facilitating intraprotozoan survival.

## GENOME ANNOUNCEMENT

Shewanella spp. are Gram-negative aquatic bacteria, ranging in length from 2 to 3 µm, and are motile by a single polar flagellum (1). Shewanella sp. CP20 was isolated from coastal waters off the coast of Clovelly, Australia (33.9121°S, 151.2629 E). Shewanella sp. CP20 survives digestion by the bacteriovorous ciliate *Tetrahymena pyriformis*. The bacterial cells that are packaged within food vacuoles are eventually expelled into the external milieu without being digested. The *Gammaproteobacteria*, to which Shewanella sp. CP20 belongs, contains many bacterial species that can survive intracellularly within a range of protozoa, e.g., Salmonella spp. (2), Yersinia spp. (3), Vibrio spp. (4), and Legionella spp. (5). The mechanisms that facilitate survival in the expelled vacuoles are not fully understood; therefore, it is important to investigate the genome of this isolate to gain insight into the genes that potentially facilitate this interaction.

The genome of Shewanella sp. CP20 was sequenced on the Illumina Hiseq 2000 platform at the Singapore Centre on Environmental Life Sciences Engineering (SCELSE), Nanyang Technological University, Singapore. A total of 1,502,730 paired-end reads (150 bp in length) were assembled into a draft genome consisting of 41 contigs with the use of Velvet sequence assembly software, version 1.2.10 (EMBL-EBI). The *N*_50_ score of the assembly was 743,057, with the length of the longest contig being 1,147,437 bp. The contigs were then submitted to the Rapid Annotation using Subsystem Technology server (RAST) (6). Initial prediction of open reading frames (ORFs) was performed by GLIMMER2, which provides identification of sequences universal in bacteria (7). Hierarchical clustering and analysis of the clusters of orthologous groups (COGS) with other members of the Shewanella genus available in the RAST database indicated that Shewanella sp. CP20 is most closely related to Shewanella loihica PV-4 (score 538) and Shewanella piezotolerans WP3 (score 506).

The Shewanella sp. CP20 genome is a circular chromosome of 4,475,402 bp with a total of 3,798 predicted coding sequences (CDSs). There was no evidence for a plasmid; however, phage DNA synthesis genes were present. The majority of the CDSs encoded genes for amino acid synthesis and degradation (354 genes), carbohydrate transport (276 genes), and the synthesis of cofactors, vitamins, prosthetic groups, and pigments (261 genes). There were 1,794 CDSs that were not assigned to a functional COG, with the majority of these being hypothetical proteins.

The annotation of the Shewanella sp. CP20 genome also identified genes associated with establishing and maintaining intraprotozoan survival. These properties included phospholipid metabolism, secretion systems for the translocation of effector proteins into the host, metal homeostasis, oxidative stress, and acid stress tolerance. The genome information obtained from Shewanella sp. CP20 will be an important addition to the understanding of intraprotozoan survival of bacteria in the marine environment.

### Nucleotide sequence accession number.

The genome sequence of Shewanella sp. CP20 has been deposited in the GenBank database under the accession number JPII00000000.
